# The Dynamics of Creative Ideation: Introducing a New Assessment Paradigm

**DOI:** 10.3389/fpsyg.2018.02529

**Published:** 2018-12-11

**Authors:** Baptiste Barbot

**Affiliations:** ^1^Department of Psychology, Pace University, New York City, NY, United States; ^2^Child Study Center, Yale University, New Haven, CT, United States

**Keywords:** ideation processes, measurement, divergent thinking, creativity, ideation ability, microdevelopment, assessment methods, digital assessment

## Abstract

Despite six decades of creative cognition research, measures of creative ideation have heavily relied on divergent thinking tasks, which still suffer from conceptual, design, and psychometric shortcomings. These shortcomings have greatly impeded the accurate study of creative ideation, its dynamics, development, and integration as part of a comprehensive psychological assessment. After a brief overview of the historical and current anchoring of creative ideation measurement, overlooked challenges in its most common operationalization (i.e., divergent thinking tasks framework) are discussed. They include (1) the reliance on a single stimulus as a starting point of the creative ideation process (stimulus-dependency), (2) the analysis of response quality based on a varying number of observations across test-takers (fluency-dependency), and (3) the production of “static” cumulative performance indicators. Inspired from an emerging line of work from the field of cognitive neuroscience of creativity, this paper introduces a new assessment framework referred to as “Multi-Trial Creative Ideation” (MTCI). This framework shifts the current measurement paradigm by (1) offering a variety of stimuli presented in a well-defined set of ideation “trials,” (2) reinterprets the concept of ideational fluency using a time-analysis of idea generation, and (3) captures individual dynamics in the ideation process (e.g., modeling the effort-time required to reach a response of maximal uncommonness) while controlling for stimulus-specific sources of variation. Advantages of the MTCI framework over the classic divergent thinking paradigm are discussed in light of current directions in the field of creativity research.

## Measuring Creative Ideation: History and Prospects

Creative Ideation (CI) refers to the process of generating original ideas in response to given open-ended problems (e.g., Fink and Benedek, 2014). CI has a rich tradition of empirical research tracing back to the 19th Century, with notably the experimental work of Alfred Binet and the Associationist school (Barbot and Guignard, 2019). But the most influential advances in the measurement of CI emerged after Guilford’s (1950) seminal push to study creativity. Since then, CI is dominantly operationalized by measures of Divergent Thinking (DT) (Kaufman et al., 2008). A classic DT task is the Alternate Uses Task (AUT, Guilford, 1967), in which respondents have to generate many uncommon uses for a common object in a limited time (e.g., a “brick” or a “newspaper”). Individual differences in the number (*Fluency*), relative uncommonness (*Originality*), and diversity (*Flexibility*) of the responses are used to characterize (cumulative) DT performance. This task-design is applicable in various modalities of responses, but verbal and figural tasks are most common given the limited domain-content knowledge they require (Barbot et al., 2015).

Since the emergence of the field of neuroscience of creativity (Fink et al., 2007) in the past few decades, CI research is increasingly (re)focusing on process-oriented and dynamic aspects (Christoff et al., 2016; Hass, 2017; Marron and Faust, 2019). Pioneering work in this line analyzed free-association switches using idiographic methods (Binet, 1900), real-time associative sequences measured by kymographic recordings (Bousfield and Sedgewick, 1944), or relations between ideas’ uncommonness and response time (Christensen et al., 1957). Presently, similar questions are tackled by coupling CI tasks with think-aloud protocols (Gilhooly et al., 2007; Pringle and Sowden, 2017), eye-tracking (Jankowska et al., 2018), systematic observation (Barbot and Lubart, 2012), or log-analysis of digital assessments (Hart et al., 2017; Loesche et al., 2018; Rominger et al., 2018). All of these methods attempt to track the chronology of CI, partition-out distinct activities (generating versus producing ideas) and capture their neurocognitive underpinning (Ellamil et al., 2012; Boot et al., 2017; Rominger et al., 2018; Benedek et al., in press).

While recent CI studies have made considerable advances in that direction by identifying the dual-process (Nijstad et al., 2010; Sowden et al., 2015) and collaborative nature of brain networks contributing to distinct cognitive resources of CI (Beaty et al., 2015; Volle, 2018), their conclusions converge with pioneering behavioral work. That is, CI is an *effortful* process (Binet, 1900; Christensen et al., 1957; Parnes, 1961; Jaušovec, 1997; De Dreu et al., 2008; Green et al., 2015; Kenett, 2018) likely more fruitful with greater *processing* *speed* in a given unit of time (Dorfman et al., 2008; Vartanian et al., 2009; Preckel et al., 2011; Forthmann et al., 2018a). For example, serial-order effect research shows that as people iterate ideas in DT-type tasks, response rates decrease while response originality increases (Christensen et al., 1957; Beaty and Silvia, 2012; Heinonen et al., 2016; Silvia Paul et al., 2017; Wang et al., 2017; Acar et al., 2018). In short, it takes more “*effort-time*” to come up with an uncommon idea (i.e., involves more exploration/thinking time) than a common one (Beaty and Silvia, 2012; Acar and Runco, 2014; Kenett et al., 2015; Wang et al., 2017; Kenett, 2018).

Because these dynamic aspects seem particularly robust (e.g., Beaty and Silvia, 2012), assessments designed to directly measure them at the person-level are needed (Hart et al., 2017; Hass, 2017; Jankowska et al., 2018; Loesche et al., 2018). Time-based measurement approaches hold great promise toward this end (Hass, 2015; Sowden et al., 2015) and could address questions such as: What is the “baseline” effort-time provided by a person in generating ideas across a range of situations? How much additional effort-time does this person need to engage in producing responses of greater originality? How is this person’s CI performance impeded by cognitive fatigue or stimuli characteristics? Before attempting to steer the classic DT assessment’s status quo in this direction, it is essential to examine its limitations and most promising variants.

## What Diverged in Divergent Thinking Tasks?

CI assessment has been somewhat “fixated” on the classic DT paradigm (Barbot, 2016). Even “gold standard” measures (e.g., Torrance’s, 2008, TTCT) still suffer from a number of task-design and psychometric shortcomings, which challenge notably the developmental study of CI (Barbot et al., 2016c). Psychometric limitations of classic DT tasks are amply documented (Plucker, 1999; Runco, 2010; Barbot et al., 2011; Zeng et al., 2011; Said-Metwaly et al., 2017). Shortcomings of their task-design framework are far less discussed and briefly outlined here.

### Stimulus-Dependency

Test-takers usually perform very differently when completing seemingly identical DT tasks that use different stimuli (e.g., AUTs of a “brick” versus “newspaper”). Almost as if they had “preferences” for one stimulus over another. Previous experience (Runco et al., 2006), tasks instructions (Nusbaum et al., 2014) or stimulus salience (Chrysikou et al., 2016; Forthmann et al., 2016) contribute to these inconsistencies, translating in heterogeneous performance across DT tasks, particularly across domains (Baer, 2012; Barbot and Tinio, 2015; Barbot et al., 2016a). Indeed, *Fluency* inter-correlations often fall on the 0.30–0.40 range, and up to 50% of fluency’s variance represents only stimulus-specific factors (Silvia et al., 2008; Barbot et al., 2016a). Such low level of alternate-form reliability is traditionally unacceptable in common psychometric standards. Although this issue was outlined since decades (e.g., Harvey et al., 1970), researchers generally underestimate how DT performance is dependent upon the stimuli at hand (Barbot et al., 2016a). Regardless, a critical feature of reliable CI measures is to sample a variety of stimuli (rather than a single one), as conducted in some DT task variants (e.g., Guilford and Hoepfner, 1966; Folley and Park, 2005; Chrysikou et al., 2016).

### Response Quality and Fluency-Dependency

Classic DT tasks first focus on the *quantity* of responses generated in a given time (e.g., 10 min). The divergent production can then be characterized *qualitatively* (e.g., *uncommonness*, *flexibility*). Hence, *fluency* is inherently confounded in summative quality scores (Forthmann et al., 2018b), with fluency-originality inter-correlations often exceeding the 0.80 range (Said-Metwaly et al., 2017). Solutions to overcome this lack of discriminant validity include (1) ignoring response quality (e.g., Batey et al., 2009; Lubart et al., 2011), (2) partialling-out the effect of fluency on quality scores (statistically or by averaging the level of *uncommonness* across all responses), or (3) relying on subjective ratings of responses’ quality (Harrington, 1975; Silvia et al., 2009). Nonetheless, the DT task format leads by default to an unequal number of responses across test-takers, from which response quality scores will be derived. As such, those with lower fluency have less opportunities to “demonstrate” their originality (impacting simultaneously the reliability of quality scores).

### Static Cumulative Performance Scores

Summary DT scores are not able to capture (and may even obscure) the dynamic processes involved in CI (Hass, 2017). In keeping with serial-order research, it could be assumed that a focus on the sequence of DT responses could address this issue (e.g., Hass, 2017). This supposes that DT responses directly transcribe the process of the thought, as if *responses* were reported at the same time as *ideas* emerge. But beyond ideas generation, it is established that (1) DT involves a monitoring and selection of ideas (e.g., Nijstad et al., 2010), and (2) during the task time, those selected ideas must be produced and refined. This has several consequences with respect to DT performance scoring: (1) response-level analysis may not accurately capture the time-course of CI; (2) factors independent from CI are indiscriminately incorporated into summative (fluency) scores (e.g., typing time necessary to produce the response; Forthmann et al., 2017); (3) originality of observable *responses* might not properly represent the originality of all *ideas* generated. These points also outline the challenge of Fluency-Originality trade-off (e.g., Fulgosi and Guilford, 1968) according to which, DT tasks’ time constraints lead test-takers to necessarily emphasize response *quantity* over *quality*, or reciprocally. Irrespective of one’s trade-off, a varying number of qualitatively heterogeneous responses (e.g., varying originality) will ultimately be aggregated into cumulative fluency and originality scores. This, in turn, provides little insight on both “baseline” levels and dynamic processes of a person’s CI.

## MTCI Framework

Most limitations outlined above can be addressed with the Multi-Trial Creative Ideation (MTCI) framework presented here. An essential feature of MTCI tasks is their use of a well-defined set of trials, each presenting a different stimulus (e.g., 20 AUT “trials”), from which a single original idea must be provided (self-paced format). Close monitoring of behavioral activity during task-resolution is used to segment response processes (e.g., isolate “think time” versus production time), and derive both cumulative and dynamic indicators of CI (e.g., baseline effort-time across trials, or incremental effort-time required to produce responses of maximal originality). Specific task-format and scoring features of the MTCI framework are now presented in greater length.

### Trials Characteristics

**FIGURE 1:**
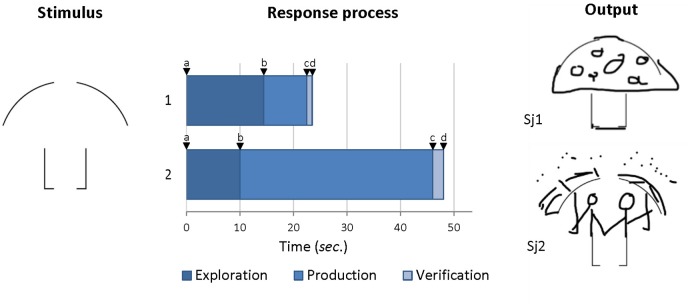
Sample item, response process times and response outputs for two subjects. In this sample trial, test-takers are required to generate an original doodle that uses the stimulus design as part of their answer (output). The response process represents the time-segmentation of the item resolution derived from log-analysis of test-takers’ interactions with the digital-platform. Phases of responses are segmented according to timestamps a to d (see description in text).

Contrary to classic DT tasks relying on a *single* stimulus that initiates *multiple* CI iterations (e.g., generating original doodles using the same abstract design over and over as starting point; See Figure 1’s stimulus), the MTCI framework requires the use of *multiple* stimuli, preferably controlled for perceptual characteristics (e.g., semantic or morphological). For each trial, a *single* response will be generated. This format resembles recent DT tasks’ adaptation for neurophysiological studies involving extensive short time-locked CI trials (Benedek et al., in press). In MTCI, this feature is proposed in the intent to (1) limit stimulus-dependency (range of stimuli offered), and (2), control the number of responses generated (addressing fluency-originality dependency and trade-off; Zarnegar et al., 1988). Although such multi-trial single-response formats showed high reliability, predictive validity (Prabhakaran et al., 2014) and convergent validity with multi-response tasks (Perchtold et al., 2018), it has been criticized for its loss of open-endedness and potential for tracking iterative CI processes (Mouchiroud and Lubart, 2001; Hass, 2017). Yet, while both formats engage DT, observable *responses* uncover only one’s reported *ideas* which, as noted above, is insufficient to genuinely track the time-course of CI. Finally, because “*problem-solving proficiency in the real world is probably a function of the number and qualitative excellence of initially generated approaches and ideas*” (Harrington, 1975, p.434), it is thought that capturing the “baseline” ideational outputs across multiple CI trials will offer (3) more engaging tasks, and (4) more ecologically valid performance scores (Kaufman and Beghetto, 2009; Forthmann et al., 2018b).

### Response Time as a Measure of Fluency

Guilford (1950) rationalizes the concept of ideational fluency by stating that “*the person who is capable of producing a large number of ideas per unit of time, other things being equal, has a greater chance of having significant ideas*” (p.452). Conceptually, this *number of ideas per unit of time* can be fairly captured with the number of responses produced in a given set time (fluency in classic DT tasks). It can also be approximated by measuring the time taken to generate a response. In MTCI, this is the only way to do so given the standardization of the number of responses produced. Of course, both approaches add-up reciprocally. For example, if one takes an average of 50 s to produce a response, it can be inferred that the equivalent fluency score for a 10 min DT-task would be 12 (assuming a constant response rate; Christensen et al., 1957). Reciprocally, a test-taker generating 15 responses in 10 min, has an average response time of 40 s. Operationalizing fluency as response time has the clear advantage of relaxing constraints of time limits for task completion (although instructions should encourage the prompt resolution of the task). Because time pressure impacts response quality in CI tasks (Runco and Acar, 2012; Forthmann et al., 2018a), such self-paced format is desirable (Kogan, 2008). It offers a naturalistic and ecologically valid setting, and provides more room for persistence (*effort*) which is an essential pathway to achieving creative ideas (De Dreu et al., 2008; Nijstad et al., 2010). Of course, this format doesn’t prevent one from “rushing through” the task instead of using time efficiently to develop responses of high originality. But this can be made visible by modeling the *effort-time* effectiveness as described below (see “Dynamic indicators of CI”).

### Administration Modality

The MTCI framework is best suited for implementation on digital-assessment platforms (e.g., Pretz and Link, 2008) that accurately monitor response time. In addition to practical advantages, digital-assessments offer a unique opportunity to unobtrusively record process data (log-analysis) inferred from the interactions between the test-taker and the digital environment (Zoanetti, 2010). Log data can be further analyzed to capture person-level dynamic markers of the task resolution process (Barbot and Perchec, 2015). While DT tasks’ implementation on computerized platforms have shown no detrimental effects on performance over paper-pencil formats (Lau and Cheung, 2010; Hass, 2015), the self-paced nature of MTCI tasks is likely more suitable than DT tasks (use of count-down) for unsupervised, non-lab-based online assessment.

### Response Process Markers

As outlined above, much of the time devoted to producing a *response* in DT tasks is not solely ideation time (e.g., Forthmann et al., 2017). Neuroscience studies have often adapted DT tasks in a way that separates CI from response production time generally confounded in DT scores (Benedek et al., in press). Regrettably, it has resulted in overly constrained paradigms, imposing rigid time-structures for different phases of CI (e.g., 15 s “think time”, 10 s response time; Ellamil et al., 2012; Perchtold et al., 2018; Rominger et al., 2018) or requiring subjects to actively “signal” an idea (Heinonen et al., 2016; Boot et al., 2017). Consistent with recent computerized assessments (Hart et al., 2017; Loesche et al., 2018), log-analysis of test-takers’ interactions with MTCI tasks can inform a more realistic chronology of broad, qualitatively distinct phases of CI (Figure 1): (1) *Exploration* – response formulation, or “thinking” phase, measured by the time between stimulus presentation (timestamp a) and the onset of the response marked by the first interaction with the digital-platform (e.g., screen-touch, or typing; timestamp b) – (2) *Production*: response production phase, measured by the time between the first (timestamp b) and the last (timestamp c) interaction with the platform in producing the response (e.g., finger-doodling for graphic responses, typing text for verbal responses) – (3) *Verification*: “control” phase in which the produced response is being validated or discarded, measured by the time between the last interaction to produce the response (timestamp c), and the action (e.g., click) to validate the response/move on to next item (timestamp d).

As illustrated (Figure 1), subject Sj1 took a total of 23 s to complete the response “mushroom,” whereas Sj2 took a total of 48 s to complete the response “singing in the rain”^1^. According to the classic DT paradigm, Sj1 would be considered more fluent (about 26 responses in 10 min assuming constant CI rate), compared to Sj2 (about 12 responses in 10 min). However, MTCI should essentially focus on *Exploration*, the principal phase during which CI operations happen (e.g., combination, idea selection), as similarly operationalized in neuroscience paradigms (Ellamil et al., 2012; Rominger et al., 2018). Accordingly, the time-analysis suggests that Sj2 spent greatest time to produce the response, which should not be confounded with CI time (*Exploration*). *Production* time – devoted to actually converting the selected idea into a response (e.g., making a doodle, or typing a response) – doesn’t inform much about the relative effort taken in generating new ideas (CI). It reflects the time engaged in elaborating the response output, as well as technological or “domain-fluency” that impacts classic DT scores (Forthmann et al., 2017). Eliminating *Production* time and focusing on *Exploration* only reveals that Sj2 was faster to come-up with the response (10 s) compared to Sj1 (14 s). The MCTI framework would therefore consider Sj2 more fluent than Sj1.

In MTCI, *Production* is cleanly partitioned-out from *Exploration*, and fine-grained information on responses’ elaboration and domain-fluency can further be derived. Log-analysis could extract information on pixel completeness of Sj1 and Sj2’s responses and corresponding action counts (elaboration), and relative speed of execution (domain-fluency). Finally, the *Verification* phase could document Sj1 and Sj2’s tendency to favor quality (e.g., closely assessing the final product) over fluency (e.g., quickly moving on to the next trial). This tendency may be at play in a fuzzier way during other phases of CI (in particular, *Exploration*). In fact, similar to neuroscience paradigms (Benedek et al., in press), it must be acknowledged that much of the specific operations happening within each phase cannot be fully deciphered using log-analysis. However, such analysis provides a much more accurate picture of the relative effort-time devoted distinctly to generating, producing and evaluating responses, compared to the cumulative DT fluency score that aggregates all three phases across all DT iterations.

### Dynamic Indicators of CI

**FIGURE 2:**
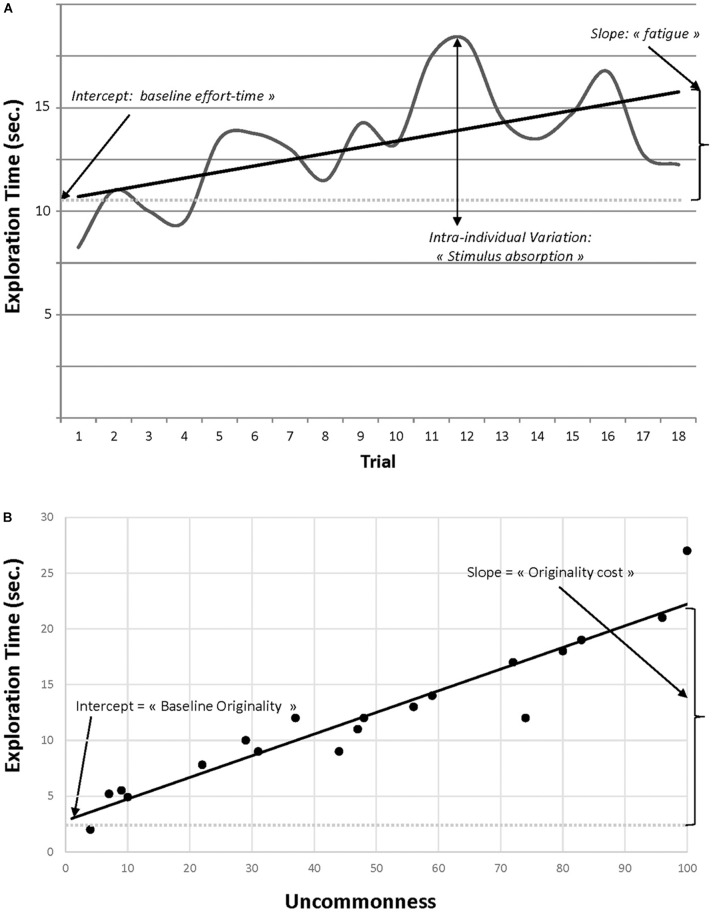
Microdevelopmental trajectories of response times across trials **(A)** and across uncommonness **(B)**. In panel **(B)**, exploration times for each trial are reorganized by order of uncommonness.

Extending the above sample item to a multi-trial context provides a number of advantages over classic DT tasks. First, MTCI’s allow one to fairly examine internal consistency of (cumulative) process times indicators (e.g., Prabhakaran et al., 2014) and uncommonness/originality ratings across trials (which DT tasks cannot, due to the unequal number of responses across test-takers). The MTCI framework also offers an unique opportunity to track intra-individual variations in performance across trials, providing a dynamic view of the CI process (Hass, 2017; Jankowska et al., 2018). Figure 2A represents Sj2’s microdevelopmental trajectory of *Exploration* time across 18 trials. Controlling for responses’ uncommonness and items difficulty, the overall performance can be characterized by a growth function with parameters meaningfully interpretable at the person-level, including an intercept (i.e., baseline *effort-time* in Exploration) and a slope (i.e., relative *fatigue* in the task resolution; see (Hass, 2017; Acar et al., 2018). Deviations from the growth function can also be fairly analyzed (e.g., capturing *stimulus absorption*, namely the person’s “preference” for one CI stimulus over another, likely to cause the stimulus-dependency challenge in DT tasks; (Barbot et al., 2016a). Extensions of trial-by-trial latent growth curve models for microdevelopmental data (Barbot et al., 2016b) could nicely accommodate such effort, while further controlling for stimulus-dependency (e.g., “method” factors by type of stimulus; Grimm et al., 2009).

Finally, a cornerstone of MTCI is that fluency shouldn’t be interpreted “in a vacuum”: In Figure 1, Sj1’s response (“mushroom”) is likely more obvious than Sj2’s response. By incorporating *Exploration* time with the corresponding *Uncommonness* of the response, and all other things being equal, MTCI would suggest a greater CI (effort-time effectiveness) for Sj2. In practice, MTCI data could help modeling this effort-time effectiveness by reordering each item-level exploration time data on a continuum of response uncommonness (Figure 2B). A person’s MTCI responses’ set should naturally show variability in uncommonness across trials. Once ranked, they provide the basis for modeling both the *baseline CI effort* (time required to come-up with the most obvious response, as captured by the growth function’s intercept) and the *originality cost* (growth function’s slope, representing the additional effort-time required to produce an idea of incremental uncommonness).

## Conclusion

Classic DT tasks have a major benefit: they have helped creativity researchers study ideation for over half a century when few alternatives were available in their toolbox. However, a shift in assessment paradigm is overdue given critical shortcomings of these tasks, preventing the accurate study of CI, its dynamics and development. This paper introduced a new CI assessment framework coined “Multi-Trial Creative Ideation” (MTCI). MTCI capitalizes on the tools of our digital era (log-analysis of interactions with digital assessments) to shift the classic DT-framework’s focus on the number of responses produced, toward a precise measure of time engaged in the production of CI outputs. This framework is thought to minimize the influence of stimulus-dependency and fluency-dependency effects, while improving CI scores’ reliability (multi-trial), ecological and external validity. It also offers the possibility to examine CI under a more dynamic lens, which aligns well with current research efforts in the field. Ongoing work and publications to follow will provide further proofs-of-concept of the key features and advantages of the MTCI framework outlined here, to pave the way for a new era of CI research and tools.

## Author Contributions

The author confirms being the sole contributor of this work and has approved it for publication.

## Conflict of Interest Statement

The author declares that the research was conducted in the absence of any commercial or financial relationships that could be construed as a potential conflict of interest.

## Footnotes

1 All subjects and data presented in this work are fictive and used for illustrative purpose only. In this example, titles could of course be typed in after all CI trials be completed.
